# Unacylated ghrelin promotes adipogenesis in rodent bone marrow via ghrelin *O*-acyl transferase and GHS-R_1a_ activity: evidence for target cell-induced acylation

**DOI:** 10.1038/srep45541

**Published:** 2017-03-31

**Authors:** Anna L. Hopkins, Timothy A. S. Nelson, Irina A. Guschina, Lydia C. Parsons, Charlotte L. Lewis, Richard C. Brown, Helen C. Christian, Jeffrey S. Davies, Timothy Wells

**Affiliations:** 1 Neuroscience & Mental Health Research Institute, and School of Biosciences, Cardiff University, Museum Avenue, Cardiff, CF10 3AX, UK; 2 Department of Physiology, Anatomy and Genetics, University of Oxford, Oxford, OX1 3QX, UK; 3 Institute of Life Science, School of Medicine, Swansea University, Swansea, SA2 8PP, UK

## Abstract

Despite being unable to activate the cognate ghrelin receptor (GHS-R), unacylated ghrelin (UAG) possesses a unique activity spectrum that includes promoting bone marrow adipogenesis. Since a receptor mediating this action has not been identified, we re-appraised the potential interaction of UAG with GHS-R in the regulation of bone marrow adiposity. Surprisingly, the adipogenic effects of intra-bone marrow (ibm)-infused acylated ghrelin (AG) and UAG were abolished in male GHS-R-null mice. Gas chromatography showed that isolated tibial marrow adipocytes contain the medium-chain fatty acids utilised in the acylation of UAG, including octanoic acid. Additionally, immunohistochemistry and immunogold electron microscopy revealed that tibial marrow adipocytes show prominent expression of the UAG-activating enzyme ghrelin O-acyl transferase (GOAT), which is located in the membranes of lipid trafficking vesicles and in the plasma membrane. Finally, the adipogenic effect of ibm-infused UAG was completely abolished in GOAT-KO mice. Thus, the adipogenic action of exogenous UAG in tibial marrow is dependent upon acylation by GOAT and activation of GHS-R. This suggests that UAG is subject to target cell-mediated activation – a novel mechanism for manipulating hormone activity.

Since its discovery^1^, the gastric hormone ghrelin has become established as a key player in the team of hormones co-ordinating central and peripheral processes with nutritional status^2^,^3^,^4^. Ghrelin is functionally unique, being the only identified peripheral signal of insufficient energy intake^5^ that promotes feeding behaviour^6^,^7^. However, ghrelin is also structurally unique, requiring post-translational acylation of the third (serine) residue to enable it to bind to and activate its cognate receptor, the growth hormone secretagogue receptor, GHS-R_1a_^1^,^8^. This modification is accomplished by the action of ghrelin *O*-acyl transferase (GOAT), a member of the membrane-bound O-acyl transferase (MBOAT) family of enzymes^9^,^10^.

Despite these landmark discoveries, one major aspect of ghrelin physiology remains unresolved: the mechanism of action of unacylated ghrelin (UAG), the predominant form of the hormone in the circulation^11^. Although lack of acylation precludes the binding of UAG to GHS-R_1a_^1^ and prevents subsequent receptor activation^8^,^11^, UAG possesses a distinct profile of biological actions. For example, like acylated ghrelin (AG), UAG suppresses post-pubertal gonadotrophin secretion^12^,^13^, reduces gastric emptying^14^ and promotes bone marrow adipogenesis^15^. However, unlike AG, UAG does not stimulate GH secretion^1^, and may even suppress skeletal growth^16^. Although controversial^17^, the majority of studies report that, unlike AG, UAG does not promote feeding behaviour^14^,^16^ or increase intra-abdominal fat mass^18^. This evidence has led several groups to speculate that UAG may exert this unique profile of actions via a novel receptor^15^,^19^,^20^, but no candidate receptors have emerged^21^.

We have, therefore, tested an alternative hypothesis, *viz* that the actions of UAG are mediated by GHS-R_1a_ after local acylation by GOAT. We have utilised a wide range of experimental approaches to test this hypothesis, examining the effect of UAG on marrow adiposity in rodents. Marrow adipocytes are particularly well suited for this study because they can be treated *in vivo* in isolation from systemic influences^15^,^22^, they respond equally to AG and UAG^15^ and can be quantified readily in excised bone. Our results suggest a novel endocrine mechanism of target cell-mediated transacylation.

## Results

### Study 1: The effect of an ibm ghrelin/UAG infusion on marrow adiposity in GHS-R-null mice

**Figure 1:**
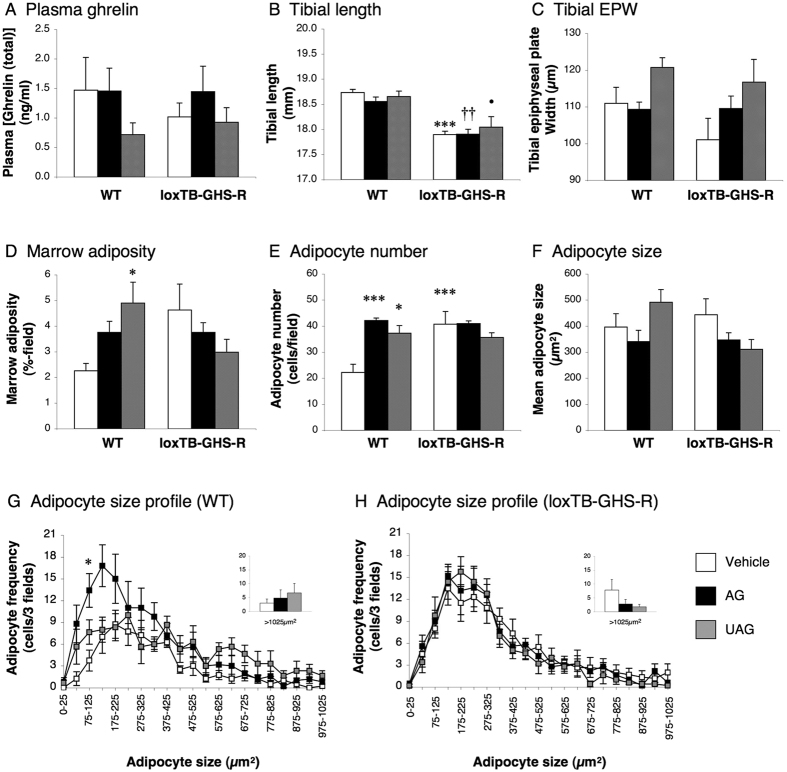
The adipogenic effect of AG and UAG is abolished in GHS-R-null mice. Quantification of circulating ghrelin (total) (**A**), tibial length (**B**), tibial epiphyseal plate width (EPW; **C**), total adiposity (**D**), adipocyte number (**E**) mean adipocyte size (**F**) and adipocyte size frequency (**G**,**H**) in mid-diaphyseal tibial marrow in male WT and loxTB-GHS-R mice receiving a 1-week intra-bone marrow infusion of vehicle (0.5 μl/hr), AG (720 ng/day) or UAG (720 ng/day). Values shown are mean ± SEM (n = 4 (vehicle), 5 (UAG) & 6 (AG)) with statistical comparisons performed by 1-way ANOVA and Bonferonni’s selected pairs *post hoc* test (*P < 0.05; ***P < 0.001 vs WT/vehicle-treated; ††*P* < 0.01 vs WT/AG-treated; •*P* < 0.05 vs WT/UAG-treated).

To investigate whether the adipogenic activity of AG and UAG in tibial marrow is mediated by GHS-R, we performed an ibm infusion of these two peptides in wild-type (WT) and loxTB-GHS-R (GHS-R-null) littermate mice. This approach revealed that infusion of AG or UAG had no significant effect on circulating ghrelin (total) in either WT or GHS-R-null mice (Fig. 1A). Similarly, although GHS-R-null mice showed a 3–4% reduction in tibial length (*P* < 0.001; Fig. 1B), neither AG nor UAG treatment had any effect on either tibial length (Fig. 1B) or tibial epiphyseal plate width (EPW; Fig. 1C). However, AG and UAG increased adipocyte number in WT mice by 90% (*P* = 0.0003; Fig. 1E) and 68% (*P* = 0.0128; Fig. 1E) respectively, though this only resulted in a significant elevation in overall adiposity in UAG-treated mice (116% increase, *P* = 0.0196; Fig. 1D). Mean adipocyte size was not significantly affected by either treatment in WT mice (Fig. 1F), but spectral analysis revealed that AG induced an increase in the population of smaller (75–125 μm^2^) adipocytes (*P* < 0.05; Fig. 1G). Although vehicle-treated GHS-R-null mice showed an 83% elevation in marrow adipocyte number in comparison to vehicle-treated WT mice (*P* < 0.001; Fig. 1E), neither AG nor UAG had any effect on any of the parameters of marrow adiposity in these animals (Fig. 1D–F,H). Indeed, the adipocyte size spectra were virtually super-imposable with that in vehicle-treated GHS-R-null mice (Fig. 1H).

### Study 2: Substrate availability in tibial bone marrow adipocytes

**Figure 2:**
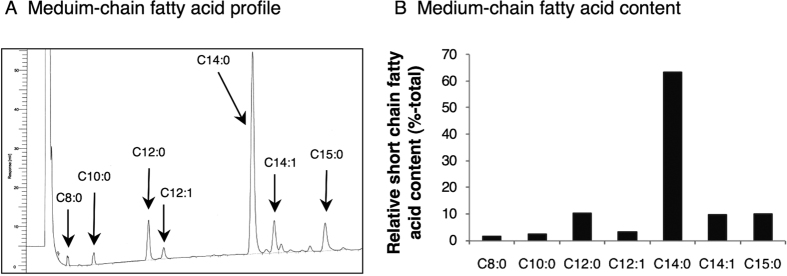
Tibial bone marrow adipocytes contain medium-chain fatty acids. Gas chromatography was used to generate a chromatogram of medium-chain fatty acids in isolated rat tibial marrow adipocytes (**A**) and the relative proportion of specific medium-chain fatty acids (**B**).

Given that UAG cannot bind to or activate GHS-R, study 1 suggests that the adipogenic action of UAG may depend upon prior conversion to AG, a process requiring the presence of appropriate medium-chain fatty acid substrates. To investigate whether marrow adipocytes contain the appropriate medium-chain fatty acids, we performed gas chromatography on lipid extracted from isolated rat tibial marrow adipocytes. These cells contain detectable levels of C8:0 (octanoic acid), C10:0, C12:0, C12:1, C14:0, C14:1 and C15:0, with C14:0 being the most abundant and octanoic acid constituting 1.5% of total medium-chain fatty acid content (Fig. 2A and B).

### Study 3: Visualisation of GOAT expression in tibial bone marrow

**Figure 3:**
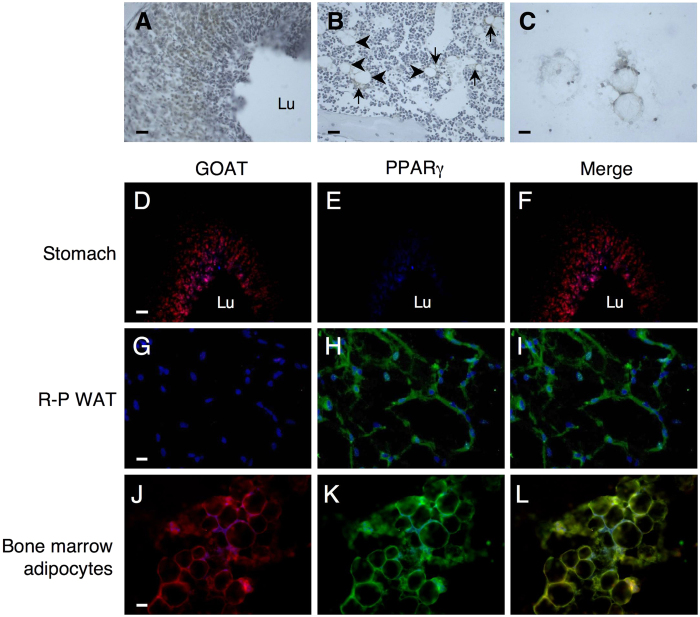
Tibial marrow adipocytes express GOAT. Visualization of GOAT expression using DAB (**A**–**C**) and fluorescence (**D**–**L**) IHC in stomach (**A**,**D**–**F**), retroperitoneal WAT (**G**–**I**) tibial bone marrow (**B**) and lipid-containing tibial bone marrow cells (**C**,**J**–**L**). In DAB images GOAT expression is shown in brown (Arrowheads (**B**) show GOAT-positive adipocytes and arrows show adjacent smaller cells). Fluorescent images (**D**–**L**) show nuclei stained with DAPI (blue), GOAT expression in red (**D**,**G**,**J**), PPARγ expression in green (**E**,**H**,**K**) and co-expression of GOAT and PPARγ in yellow in merged images (**F**,**I**,**L**). Scale bar: 50 μm (**A**,**B**,**D**–**I**), 25 μm (**C**,**J**–**L**); Lu stomach lumen.

Given the presence of the appropriate medium-chain fatty acids, we used immunohistochemistry (IHC) to investigate whether marrow adipocytes express the ghrelin-activating enzyme, GOAT. Our IHC approach, which revealed the expected visualisation of GOAT expression in the lower two thirds of the gastric pits (Fig. 3A and D), confirmed the GOAT was also expressed in mature marrow adipocytes (arrowheads in Fig. 3B) and in aggregations of smaller adjacent cells (arrows in Fig. 3B). Smears of lipid-containing rat marrow cells also showed marked GOAT expression (Fig. 3C). Double fluorescence IHC demonstrated that stomach expressed GOAT but not the adipogenic biomarker PPARγ (Fig. 3D–F). In contrast, UAG-insensitive retroperitoneal adipocytes expressed PPARγ but not GOAT (Fig. 3G–I), whereas isolated rat marrow adipocytes expressed both PPARγ and GOAT (Fig. 3J–L).

### Study 4: Sub-cellular distribution of GOAT expression in tibial bone marrow adipocytes

**Figure 4:**
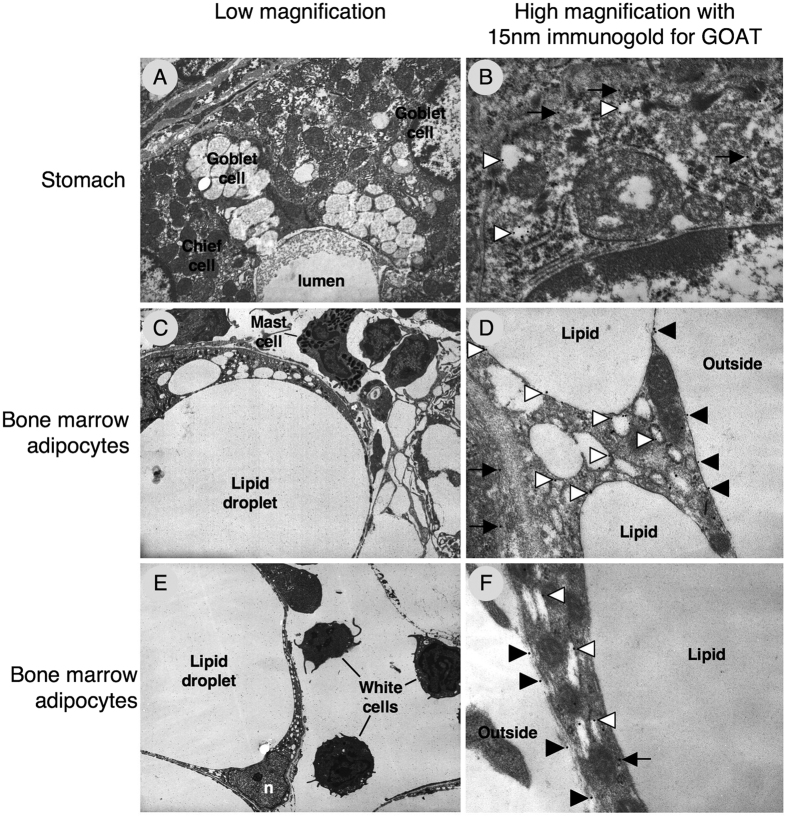
GOAT is expressed in intracellular and plasma membranes of tibial marrow adipocytes. Low magnification electron micrographs showing the general appearance of neuroendocrine cells in rat stomach (**A**) and bone marrow adipocytes (**C**,**E**). High magnification electron micrographs with immunogold labelling of GOAT in stomach (**B**) and tibial marrow adipocytes (**D**,**F**) showing expression in the cytoplasm (arrows), trafficking vesicles (open arrowheads) and plasma membrane (closed arrowheads).

We subsequently used immunogold electron microscopy to characterize the subcellular distribution of GOAT. In the neuroendocrine cells of the stomach, GOAT was expressed in the cytoplasm (arrows) and in association with the membrane of electron-translucent trafficking vesicles (arrowheads; Fig. 4B). Similarly, in the tibial marrow adipocytes, GOAT immunoreactivity was identified in the cytoplasm (arrows) and in the membrane of both large and small lipid-trafficking vesicles (white arrowheads; Fig. 4D & F). In addition, immunogold labelling for GOAT was clearly present in the plasma membrane of the marrow adipocytes (black arrowheads; Fig. 4D and F).

### Study 5: The effect of an ibm UAG infusion on marrow adiposity in GOAT-KO mice

**Figure 5:**
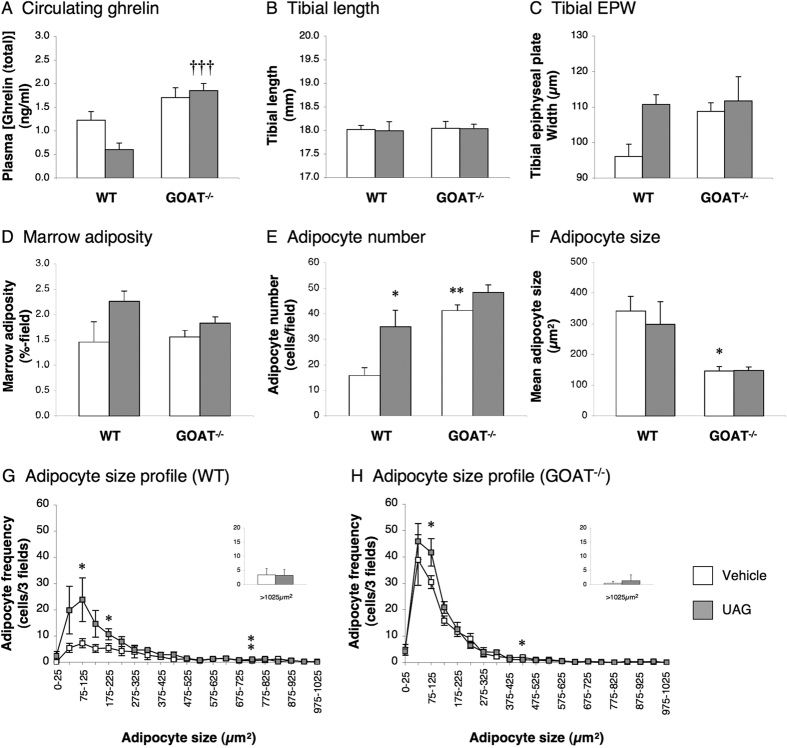
The adipogenic effect of UAG is abolished in GOAT-KO mice. Quantification of circulating ghrelin (total) (**A**), tibial length (**B**), tibial epiphyseal plate width (EPW; **C**), total adiposity (**D**), adipocyte number (**E**) and mean adipocyte size (**F**) in mid-diaphyseal tibial marrow in male WT and GOAT-KO mice receiving a 1-week intra-bone marrow infusion of vehicle (0.5 μl/hr) or UAG (720 ng/day). Comparison of the distribution profiles of adipocyte size between vehicle- and UAG-treated WT (**G**), and vehicle- and UAG-treated GOAT^−/−^ mice (**H**) are shown with adipocytes larger than 1000 μm^2^ presented as inset histograms. Values shown are mean ± SEM (n = 5 (vehicle- and UAG-treated WT, vehicle-treated GOAT^−/−^) & 6 (UAG-treated GOAT^−/−^)), with statistical comparisons performed by 1-way ANOVA and Bonferonni’s selected pairs *post hoc* test (**A**–**C**) or Student’s t-test (**G**,**H**) (*P < 0.05; **P < 0.01 vs vehicle-treated WT).

Given the presence of the elements required for the acylation of UAG, we investigated whether the adipogenic effect of UAG *in vivo* is dependent upon GOAT, by treating GOAT-KO mice with a 1-week ibm infusion of UAG. Although halved, mean circulating ghrelin in UAG-treated WT mice was not significantly different from that in vehicle-treated WTs (Fig. 5A). Deletion of *GOAT* alone had no effect on circulating ghrelin (Fig. 5A). However, although unaffected by UAG treatment, circulating ghrelin in GOAT-KO mice was three times that in UAG-infused WT mice (*P* < 0.001; Fig. 5A). Similarly, tibial length was unaffected in GOAT-KO mice and, as in study 1, ibm infusion of UAG had no statistically significant effect on either tibial length or EPW (Fig. 5B and C). The effect of UAG treatment in WT mice was also similar to that seen in study 1 above (Fig. 1D–F), adipocyte number being doubled (*P* = 0.0152; Fig. 5E), without significantly affecting mean adipocyte size or overall adiposity (Fig. 5F and D). This increase in adipocyte number was particularly prevalent in smaller adipocytes (Fig. 5G). In contrast, UAG had no effect on any of the parameters of marrow adiposity in GOAT-KO mice (Fig. 5D–F). Interestingly, in comparison to WT mice, vehicle-treated GOAT-KO mice showed a significant increase in the population of small adipocytes in tibial marrow (Fig. 5E,G and H).

## Discussion

UAG, the predominant form of ghrelin in the circulation, has been shown to exert a distinct profile of activities, but the mechanism of action of this hormone has remained enigmatic; an enigma exemplified by the adipogenic action of UAG in bone marrow. Like AG, UAG promotes marrow adipogenesis, and while it was assumed that this represented evidence of a GHS-R-independent action of UAG and possibly AG^15^, evidence for an alternative receptor mediating this action has remained elusive. We have now re-examined this phenomenon and the data we present here support the surprising hypothesis that the adipogenic action of UAG is dependent upon both GHS-R, the receptor to which it does not usually bind^8^,^11^, and the acylating activity of GOAT.

Using our miniaturised intra-bone marrow infusion strategy, we have demonstrated that the adipogenic activity of AG and UAG previously seen in rats^15^ was replicated in mice. However, we were surprised to find that the adipogenic activity of both forms of ghrelin was abolished in mice in which transcription of GHS-R is blocked. Since, the absence of the post-translational addition of an acyl side-chain precludes the binding of UAG to GHS-R^1^,^8^,^11^, we investigated the possibility that UAG could be acylated in the bone marrow milieu. Fatty acid profiling in isolated tibial marrow adipocytes revealed that these lipid-storing cells contain a range of detectable medium-chain fatty acids, including octanoic acid, the principle substrate for the activation of UAG^23^. It should be noted that while UAG can also be acylated with C10:0, C12:0, and C14:0^23^,^24^, all of which are present in marrow adipocytes, icv injections of C12:0- and C14:0-AG are effective in elevating fat mass^24^, while C10:0-AG is as effective as octanoylated ghrelin in promoting GH secretion^23^. Thus, although we have not quantified the relative utilisation of these medium-chain fatty acids in the activation of UAG in bone marrow, the substrate required for acylation is clearly present.

Given the presence of these medium-chain fatty acids, we developed an immunohistochemical approach for visualising the presence of GOAT in marrow adipocytes. This revealed co-localisation of GOAT with the adipocyte-specific marker, PPARγ in marrow adipocyte smears and prominent expression of GOAT in the aggregations of cells surrounding lipid-containing adipocytes. Whether these smaller cells represent pre-adipocytes or earlier stages in the differentiation process remains to be determined. This study clearly adds another tissue to the selective list of tissues now known to express GOAT, which includes the stomach, hypothalamus and pituitary, but not the pancreas, liver, kidney, spleen, heart, skeletal muscle, lung or WAT^25^,^26^,^27^, the latter being confirmed in the present study.

Since we have shown that these cells respond to exogenous UAG, we used the same primary antibody to characterise the sub-cellular localisation of GOAT with immunogold electron microscopy. This revealed that although some GOAT immunoreactivity was located in the cytoplasm, the majority was associated with membrane structures, as would be expected for a member of the MBOAT family^9^. Its presence in the membrane of lipid trafficking vesicles suggests a role for GOAT in the regulation of lipid storage and release, whereas its presence in the plasma membrane provides circumstantial evidence that GOAT may enable marrow adipocytes to activate and subsequently respond to exogenous UAG.

We believe our data to represent the first description of the sub-cellular localisation of GOAT, even in the stomach. GOAT has previously been located in membranes of the endoplasmic reticulum (ER)^28^ where it may transport acyl-coA from the cytosol to the ER lumen^9^, and regulate the balance between AG and UAG secretion. Although assumed to be associated with the secretory pathway, our study indicates that GOAT expression in gastric neuroendocrine cells appears to be most prominent in the membrane of electron-translucent trafficking vesicles rather than the electron-dense secretory granules. Since cultured cells require the addition of n-octanoic acid to the medium for efficient AG production^29^, it is possible that these trafficking vesicles provide the substrate necessary for the acylating action of GOAT.

To investigate whether the presence of GOAT is essential for the adipogenic activity of UAG, we repeated our tibial marrow infusion experiment in GOAT-KO mice. The UAG-induced doubling of marrow adipocyte number seen in WT mice was abolished by the deletion of GOAT. Thus, in addition to the previous evidence that co-transfection of endocrine and non-endocrine cells with GOAT and pre-proghrelin cDNAs results in detectable AG production^9^,^10^, we have demonstrated that the adipogenic effect of exogenous UAG in tibial bone marrow is dependent upon the function of GOAT and the presence of GHS-R. Whether marrow adipocytes or pre-adipocytes express pre-proghrelin themselves, enabling acylation of intra-cellular UAG, or whether the effects reported here in males are observable in females, remains to be determined.

A couple of alternative mechanistic explanations should be considered. Firstly, it is possible that a lack of AG feedback in GHS-R-null and GOAT-KO mice could result in an elevation in UAG production and a maximal adipogenic response in tibial marrow via the unidentified receptor. The doubling of the marrow adipocyte population in GHS-R-null and GOAT-KO mice, which is due to the increased prevalence of smaller adipocytes, appears to support this suggestion and could potentially mask any additional activity of exogenous UAG. However, it should be noted that circulating (total) ghrelin, the majority of which is UAG^11^, was not elevated in either the loxTB-GHS-R or GOAT-KO mice in this study. These data confirm previous reports that neither AG nor UAG are elevated in loxTB-GHS-R mice^30^,^31^ and that GOAT-KO mice display levels of circulating UAG within the physiological range^10^. Thus, although we have not quantified ghrelin production within the tibial marrow, we think this explanation to be unlikely.

In this context it should be noted that while GHS-R-null mice show relatively normal circulating IGF-1^32^, GOAT-KO mice show elevated circulating IGF-1 in conjunction with reduced GH secretion^33^. Since marrow adipocytes are exquisitely sensitive to GH^34^, a modest reduction in GH could explain the increase in adipocyte number in both of these models. That said, the elevated marrow adiposity in the profoundly GH-deficient *dw*/*dw* rat remains remarkably sensitive to the adipogenic effects of both AG and UAG^15^.

Secondly, although it was initially thought that UAG does not bind to GHS-R, subsequent studies have demonstrated that this is not the full picture. Although UAG has been shown to antagonize the metabolic effects of AG^35^ it acts as an agonist of GHS-R_1a_ but with a 1000-fold lower efficacy than AG^36^. This latter action appears to be dependent, in part, upon the electrostatic attraction of the ligand to the receptor and its associated plasma membrane^37^. However, given that AG and UAG are equipotent in stimulating marrow adipogenesis and this action is entirely dependent upon the presence of GOAT, any contribution of direct agonist activity of UAG in the current study is likely to be negligible.

Thirdly, recent evidence has demonstrated that GHS-R_1a_ forms heterodimers with a wide array of G-protein-coupled receptors, even in the absence of AG^38^. Thus, the absence of an adipogenic effect of UAG in GHSR-null mice could be explained by a lack of dimer formation with the unidentified receptor for UAG. However, our demonstration of the presence and necessity of GOAT in this action, suggests that while we cannot completely exclude the possibility that UAG may activate an unidentified dimer partner, the weight of evidence seems to favour a direct action via GHS-R_1a_.

In the context of the bone marrow, the adipocytes are emerging as more than just a space filler, as originally thought. Secreting high levels of leptin^39^, these cells are able to influence the bone microenvironment and exert an impact on the microarchitecture and mechanical integrity of bone^22^. Our data indicate that the balance between bone forming and fat storing cells in the marrow is likely to be nutritionally regulated, and that AG and UAG may defend marrow fat from utilisation during starvation^40^. However, it is also becoming clear that the relationship between marrow adipocytes and bone integrity in different bone types is not uniform^41^.

Although our evidence that the adipogenic action of UAG in bone marrow is dependent upon acylation and activation of GHS-R, this is clearly not the case for all of the actions of UAG. For example, whilst deletion of *GHS-R* abolishes the effect of icv UAG on fat mass and hyperinsulinaemia^42^, it fails to prevent the vasodilatory effects of UAG^43^, the UAG-induced impairment of fasting-induced skeletal muscle atrophy^44^ and skeletal muscle regeneration^45^, or the effects of UAG on genes involved in glucose and lipid metabolism in fat, muscle and liver^46^.

Indeed, our data lends weight to the emerging evidence that marrow adipocytes are not representative of extra-medullary adipocytes. For example, we have previously shown that while retroperitoneal WAT is the most sensitive of the intra-abdominal depots to AG exposure^15^, it fails to respond to iv infusions of UAG^18^. Our current evidence suggests that this insensitivity of abdominal WAT may be due to a lack of GOAT expression, because deletion of GHS-R abolishes the positive influence of AG on abdominal WAT mass, thereby providing functional evidence that abdominal adipocytes express GHS-R^18^.

**Figure 6:**
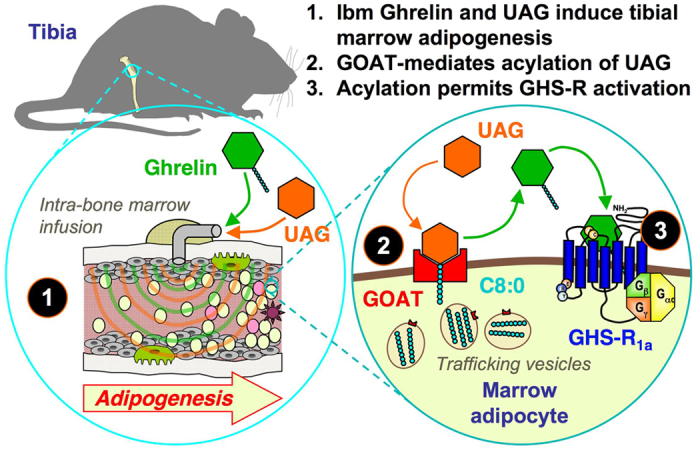
The transacylation of exogenous UAG in tibial marrow adipocytes. Intra-bone marrow (ibm) infusions of ghrelin and UAG induce adipogenesis in tibial marrow of mice. 2. Ghrelin *O*-acyl transferase (GOAT) in the plasma membrane of marrow adipocytes utilises octanoic acid (C8:0) and other medium-chain fatty acids from within marrow adipocytes to acylate exogenous UAG. 3. Acylation of UAG to ghrelin permits activation of GHS-R_1a_.

However, we believe our data to have wider significance. Not only does our evidence confirm that UAG is a significant hormone in its own right, but it implies that target cells for this hormone are able to regulate their response by manipulating the activity of incoming UAG. Our hypothesis is that expression of GOAT in the plasma membrane of cells containing substrate for acylation can add the required sidechain to exogenous UAG, thereby enabling activation of GHS-R (Fig. 6). In this model GOAT, would activate UAG in a reciprocal fashion to the role of deiodinase 2, which activates T4 by the removal of an iodine. This novel mechanism may be important in tissues known to express GOAT, including in the stomach, enteroendocrine cells of the intestine, anterior pituitary, pancreatic islets, thyroid, parathyroid^25^,^27^,^47^ and hypothalamus. Indeed, in the hypothalamus, where the uptake of UAG and AG are differentially controlled^48^, region-specific expression of GOAT may enable individual nuclei to activate GHS-R in response to UAG, but not to AG. In addition, the nutritional regulation of GOAT expression in the hypothalamus provides another layer of mechanistic complexity^27^.

In summary, we have demonstrated that in tibial bone marrow, UAG promotes adipogenesis by a mechanism involving GOAT-mediated acylation and the activation of GHS-R. Thus, access of UAG to the site of action, coupled with the presence of GOAT on the plasma membrane and the expression of GHS-R will determine the unique activity profile of this important metabolic hormone.

## Methods

### Animals

The animal procedures described, including those involving genetically modified animals, were conducted in accordance with the UK Animals (Scientific Procedures) Act, 1986 and the ARRIVE Guidelines, and were specifically approved by ethical review at Cardiff University. Male rats (Sprague Dawley (SD) (Harlan UK Ltd., Bicester, Oxon, UK); Studies 2, 3 & 4) and mice (Studies 1 & 5) used were housed under conditions of 14 h light/10 h dark (lights on at 05.00 h) in the animal facility at Cardiff University, with food (Harlan Teklad Rodent Maintenance Diet containing 4.9% oil and 14.2% protein) and water available *ad libitum*. loxTB-GHS-R mice and their wild type (WT; C57Bl6) littermate controls^32^ were bred from heterozygous x heterozygous matings in the animal facility at Cardiff University from founder stock imported from the University of Texas Southwestern Medical Center (Dallas, TX) (Study 1). Homozygous GOAT-KO mice and their WT (C57Bl6) controls^10^ were imported from Taconic Farms (Hudson, NY) (Study 5).

### Study 1: Effect of an ibm ghrelin/UAG infusion on marrow adiposity in GHS-R-null mice

9-month old male loxTB-GHS-R (GHS-R-null) mice (31.8 ± 0.8 g (n = 16)) and age-matched male WT littermate controls (34.4 ± 0.6 g (n = 13); *P* < 0.01) were prepared with right tibial intra-bone marrow (ibm) catheters connected to osmotic minipumps (Alzet model 1007D) primed to deliver vehicle (sterile isotonic saline containing BSA (1 mg/ml) and heparin (5 U/ml) at 0.5 μl/h), AG (720 ng/day) or UAG (720 ng/day) for 7 days. This approach, performed under isoflurane anaesthesia, was a miniaturised version of the technique previously used in rats^15^,^22^. This dose of ghrelin was calculated to produced a mid-range increase in bone marrow adiposity based upon on a dose-response curve for intravenously (iv) infused AG constructed in mice (Supplementary Figure 1) and the difference in effectiveness of iv and ibm infusions previously reported in rats^15^. At term, the mice were weighed, re-anaesthetized with isofluorane and decapitated. Plasma samples were separated from trunk blood and stored at −20 °C for subsequent determination of circulating ghrelin. Right tibiae were dissected, the length measured using a hand-held micrometer and stored for subsequent quantification of marrow adiposity and EPW.

### Study 2: Quantification of lipid content of marrow adipocytes

To determine whether marrow adipocytes contain the required substrate for octanoylation, the medium-chain fatty acid profile was determined in isolated rat marrow adipocytes. Three male SD rats (24 weeks-old; 424–474 g) were concussed and killed by cervical dislocation and left tibiae excised. After removal of the distal epiphyses, the marrow contents were centrifuged into sterile isotonic saline and the adipocytes aspirated from the aqueous surface and pooled. Lipid contents were extracted using the Kates method^49^ and fatty acid methyl esters prepared by transmethylation with 2.5% H_2_SO_4_ in HPLC-grade methanol/toluene (2:1) at 70 °C for 2 h. A known amount of pentadecanoate (15:0) was added as an internal standard to enable subsequent fatty acid quantification. A Clarus 500 gas chromatograph with a flame ionizing detector and fitted with a 30 m × 0.25 mm internal diameter capillary column (Perkin Elmer Elite 225) was used for fatty acid identification. Peaks were integrated using TotalChrom software (Perkin-Elmer, version 6.2.1).

### Study 3: Visualisation of GOAT expression in tibial bone marrow

An IHC approach was developed for visualizing GOAT expression in stomach (12 μm cryostat sections of fresh frozen tissue taken from the greater curvature of the corpus and stored at −20 °C until use), tibiae (7 μm longitudinal sections of decalcified, paraffin embedded tibiae, including mid-cortical marrow), tibial marrow adipocyte smears (adipocytes obtained as above (study 2) and smeared onto poly-L-lysine-coated microscope slides and stored at −20 °C) and retroperitoneal WAT (12 μm cryostat sections of fresh frozen tissue stored at −20 °C until use). These tissues were harvested from rats in study 2.

**Figure 7:**
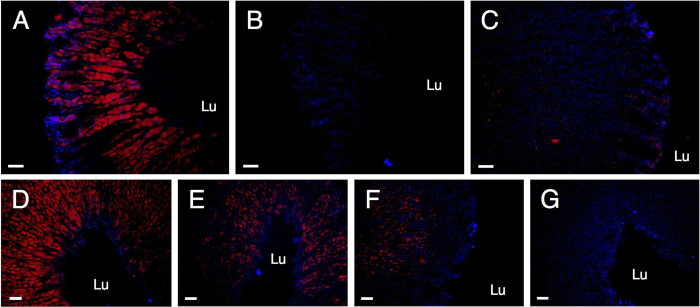
Validation of GOAT IHC in rat stomach. Fluorescent IHC for GOAT in 12 μm cryostat sections of rat stomach either with (**A**) or without (**B**) inclusion of the primary rabbit anti-GOAT antibody (1:1000 dilution), or with pre-absorption of the primary antibody (1:1000 dilution) with GOAT peptide (10 μM; **C**). Images D-G show the effect of serial dilutions of the primary antibody (**D**: 1:1000; **E**: 1:2000; **F**: 1:4000; **G**: 1:8000). Nuclei were stained with DAPI (blue), with GOAT expression seen in red. Scale bars: 50 μm; Lu stomach lumen.

Stomach sections were fixed in 4% paraformaldehyde (in PBS), subjected to antigen recovery (incubation in 10 mM sodium citrate in 0.05% Tween 20 at 60 °C for 1 hr), permeabilized in methanol, blocked in 10% normal goat serum (NGS) and incubated overnight with a rabbit anti-GOAT primary antibody; Table 1. Incubation with a fluorescent secondary antibody (1:500; Cy3-conjugated sheep anti-rabbit IgG) produced the expected^47^ red fluorescence for GOAT in the mid to lower portions of the gastric pits (Fig. 7A). The fluorescent signal was dependent upon primary antibody dilution (Fig. 7D–G) and was abolished by omission of the primary antibody (Fig. 7B) or pre-incubation of the primary antibody (1:1000 dilution) with rat GOAT peptide (10 μM; Cat no. 032-12; Phoenix Europe GmbH, Karlsruhe, Germany) (Fig. 7C). A 1:2000 dilution of the primary antibody was used in subsequent experiments.

Since paraffin-embedded tibial sections yielded significant autofluorescence, non-fluorescent IHC was used for these samples. Tibial marrow sections were de-waxed, and, together with stomach sections and tibial marrow adipocyte smears were treated as above up to and including incubation with the primary antibody, except that permeabilization with methanol was replaced with treatment with hydrogen peroxide (0.3% in methanol). Sections were incubated with a horseradish peroxidase-conjugated goat anti-rabbit secondary antibody, stained with diaminobenzidine tetrahydrochloride (DAB) and counterstained with Meyer’s haematoxylin.

GOAT-positive cells in the marrow cell smears were identified by double fluorescence IHC for GOAT and PPARγ. The above IHC protocol was followed except that the primary antibody solution contained both rabbit anti-GOAT and mouse anti-human PPARγ (1:100 dilution; Cat No 419300; Invitrogen Corporation, Camarillo, CA; Table 1) and the secondary antibody solution contained both Cy3-linked sheep anti-rabbit and Alexa-fluor488-linked goat anti-mouse antibodies (1:500).

### Study 4: Visualisation of the subcellular distribution of GOAT in tibial bone marrow adipocytes

The subcellular distribution of GOAT in marrow adipocytes was visualised by immunogold electron microscopy (EM). Isolated tibial marrow adipocytes were obtained as in study 2 from two 9 week-old male SD rats, centrifuged into sterile saline and aspirated into 2% glutaraldehyde (EM grade): 2% formaldehyde in 0.1 M phosphate buffer. After 5 mins cells were transferred into 10% fixative and stored at 4 °C prior to embedding. Cells and 0.5 mm^3^ segments of stomach were prepared by a standard method^50^ in which segments were stained with uranyl acetate (2% w/v in distilled water), dehydrated through methanol (70–100%) and embedded in LR Gold. Ultrathin sections (50–80 nm) were prepared using a Reichert ultracut S microtome and mounted on 200 mesh nickel grids. Ultrathin sections were incubated with rabbit anti-GOAT antibody (1:1000 in 0.1 M phosphate buffer containing 0.1% egg albumin) at room temperature for 2 h and with Protein A-15 nm gold complex (1:50) for 1 h. No non-specific labelling was observed in the absence of the primary antibody. After immunolabelling, sections were lightly counter-stained with lead citrate and uranyl acetate and examined with a JEOL transmission electron microscope, images being collected with a GATAN Orius camera.

### Study 5: The effect of an ibm UAG infusion on marrow adiposity in GOAT-KO mice

To establish whether local GOAT expression determines the effectiveness of UAG, 9-month old male GOAT-KO mice (31.8 ± 0.9 g) and age-matched male WT littermate controls (34.0 ± 0.9 g; *P* = 0.054) were prepared with ibm catheters and osmotic minipumps primed to deliver either vehicle or UAG as in study 1. At term, the mice were weighed, re-anaesthetized with isofluorane and decapitated. Plasma samples separated from trunk blood were stored at −20 °C for subsequent determination of circulating ghrelin and right tibiae were dissected as above for quantification of marrow adiposity.

### Quantification of marrow adiposity and EPW

Marrow adiposity and EPW were quantified as previously described^15^. Briefly, tibiae were fixed, decalcified in EDTA and sectioned as in study 3, with sections stained with Masson’s Trichrome or Toluidine Blue for quantification of EPW and marrow adiposity respectively. Leica Q-Win was used to measure EPW under light microscopy. Adiposity was quantified using the method of Gevers *et al*.^34^. Digital images (1 × 186304 μm^2^ field/section; *3* sections/tibia) were taken of mid-diaphyseal marrow with a Leica DFC300FX digital camera and a Leica DMLB microscope. Image J was used to quantify the degree of adiposity, adipocyte number and adipocyte size.

### Quantification of circulating ghrelin

Circulating (total) ghrelin was quantified in terminal plasma samples from studies 1 and 5 using a rat/mouse Ghrelin (total) ELISA kit (EZRGRT-91K; Millipore, UK) as per the manufacturer’s instructions. Absorbance was measured using a POLARstar Omega plate reader (BMG Labtech, Cambridge, UK) and concentrations determined according to the manufacturer’s instructions (Intra-assay variation: 10.96%).

### Statistical analyses

Results are expressed as mean ± SEM (with the number of animals used per group indicated in the figure legends), and differences between groups compared by Student’s t-test or one-way ANOVA followed by Bonferroni’s (selected pairs) *post-hoc* test using GraphPad Prism, with *p* < 0.05 considered significantly different.

## Additional Information

**How to cite this article:** Hopkins, A. L. *et al*. Unacylated ghrelin promotes adipogenesis in rodent bone marrow via ghrelin *O*-acyl transferase and GHS-R_1a_ activity: evidence for target cell-induced acylation. *Sci. Rep.* **7**, 45541; doi: 10.1038/srep45541 (2017).

**Publisher's note:** Springer Nature remains neutral with regard to jurisdictional claims in published maps and institutional affiliations.

## Supplementary Material

Supplementary Information

## Figures and Tables

**Table 1:** Antibodies used for IHC and immunogold EM visualization of GOAT.

Antigen	Host Species	Manufacturer	Cat #	Final Dilution
Primary Antibodies				
Human GOAT	Rabbit	Phoenix Europe GmbH	H-032-12	1:2000
Human PPARγ	Mouse	Invitrogen Corporation	419300	1:100
Secondary Antibodies (IHC)				
Cy3-conjugated anti-rabbit IgG	Sheep	Sigma-Aldrich Company Ltd.	C2306	1:500
Alexa-fluor488- anti-mouse	Goat	Life Technologies, USA	A11001	1:500
Secondary Antibodies (Immunogold EM)				
AU conjugate (Protein A 15 nm Gold)	Rabbit	British Biocell, Cardiff, UK	EM.PAG15	1:50
