# Supplementary material for: Unacylated ghrelin promotes adipogenesis in rodent bone marrow via ghrelin O-acyl transferase and GHS-R1a activity: evidence for target cell-induced acylation
Source: Sci Rep. 2017 Mar 31;7:45541. doi: 10.1038/srep45541 (PMC5374529; doi:10.1038/srep45541)
Supplement: Supplementary Information [file srep45541-s1.pdf]

Unacylated ghrelin promotes adipogenesis in rodent bone marrow via ghrelin *O*-acyl transferase and GHS-R_{1a} activity: evidence for target-cell-induced acylation

Authors:
Anna L Hopkins
Timothy AS Nelson
Irina A Guschina
Lydia C Parsons
Charlotte L Lewis
Richard C Brown
Helen C Christian
Jeffrey S Davies
Timothy Wells

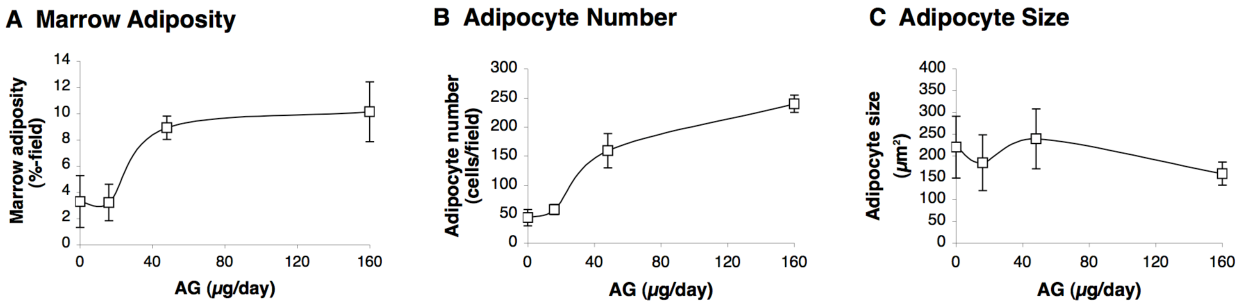

Supplementary Figure 1: The dose-dependent effect of intravenous AG infusion on tibial marrow adiposity in mice. Male C57bl6 mice (12 weeks old; 22.6-31.4g bred in the animal facility at Cardiff University) were prepared with a jugular vein catheter connected to an osmotic minipump (Alzet model 2001) primed to deliver vehicle (sterile isotonic saline containing BSA (1mg/ml) and heparin (5U/ml) at 1.0µl/h; n=4) or AG (16 (n=4), 48 (n=3) or 160 (n=2) µg/day) under isoflurane anaesthesia. After 1 week of infusion, mice were re-anaesthetised and killed by decapitation, tibiae being excised and processed for the quantification of marrow adiposity as in materials and methods.
